# Damage in the Multiethnic Malaysian Systemic Lupus Erythematosus (SLE) Cohort: Comparison with Other Cohorts Worldwide

**DOI:** 10.1371/journal.pone.0166270

**Published:** 2016-11-15

**Authors:** Syahrul Sazliyana Shaharir, Heselynn Hussein, Sakthiswary Rajalingham, Mohd Shahrir Mohamed Said, Abdul Halim Abdul Gafor, Rozita Mohd, Ruslinda Mustafar

**Affiliations:** 1 Department of Internal Medicine, Pusat Perubatan Universiti Kebangsaan Malaysia (UKMMC), Jalan Yaacob Latiff, 56000 Kuala Lumpur, Malaysia; 2 Department of Medicine, Putrajaya Hospital, Jalan P9, Presint 7, 62250 Putrajaya, Malaysia; JAPAN

## Abstract

Systemic Lupus Erythematosus (SLE) is a chronic autoimmune disease and despite the improvement in the survival in the past few decades, the morbidity due to disease damage remains significant. The objectives of this study were to investigate the disease damagepattern and determine the associated factors of damage in the multi-ethnic Malaysian SLE patients. We consecutively 424SLE patients who attended a consistent follow-up at the National University of Malaysia Medical Centre and Putrajaya Hospital were recruited. Disease damage was assessed using the SLICC/ACR (Systemic Lupus International Collaborating Clinics/American College of Rheumatology) Damage Index (SDI) scores. Information on their demographics and disease characteristics were obtained from the clinical record. Univariate analysis was performed and the best model of independent predictors of disease damage was determined by multivariate logistic regression analysis. A total of 182 patients (42.9%) had disease damage (SDI ≥1). A significantly higher number of Indian patients had disease/organ damage and they predominantly developed steroid-induced diabetes mellitus (SDM). Patients with corticosteroid-induced osteoporosis (CIOP) were more likely to be Malayswhile majority of patients who developed malignancy were Chinese (p<0.05). In the univariate and multivariate analyses, disease damage was significantly associated with age, Indian ethnicity, lower mean cumulative C3 level, neuropsychiatry lupus (NPSLE), and antiphospholipid syndrome (APLS). Patients who had ever and early treatment with hydroxychloroquine(HCQ)were less likely to develop disease damage while more patients who had received oral prednisolone ≥1mg/kg daily over 2 weeks had disease damage (p<0.05). In conclusion, there were inter-ethnic differences in the damage pattern and risks among SLE patients.

## Introduction

Systemic Lupus Erythematosus (SLE) is an autoimmune disease which is characterized by multi-system organ inflammation. Despite a remarkable improvement in the management of SLE and improved survival in the past few decades [1], however, the morbidity due to organ damage sequelae remains significant. In view of this, apart from disease activity and quality of life, measurement of disease damage is very important as part of a standard assessment in the management of SLE patients.

The Systemic Lupus Erythematosus International Collaborating Clinics/American College of Rheumatology (SLICC/ACR) damage index for SLE is a well validated tool to assess accumulated damage index (DI) since the onset of the disease [2]. The damage includes non-reversible changes in organs and systems affected by the disease process itself, its therapy, or inter-current illness. The SLICC/ACR damage index is highly reproducible and has been shown to have a good agreement with prospective and retrospective measurement of DI [3]. Disease damage measured with SLICC/ACR damage index is well correlated with mortality [4] and quality of life in patients with SLE [5]. In addition, a different pattern of organ damage such as renal, neuropsychiatry, cardiovascular and pulmonary damage also predicts poorer prognosis and mortality [2, 6].

Disease outcome in SLE is largely influenced by various factors which include genetic, socio-cultural, behavioural and environment [7]. Ethnicity was considered as part of a genetic marker and it is well established that Asian and Non-Caucasian SLE patients generally exhibit more severe lupus and poorer outcome [8–11]. Several studies have evaluated the systemic damage among SLE patients from various ethnic backgrounds. The LUMINA (LUpus in MInorities: NAture vs nurture) cohort revealed a different disease outcome and damage among their Caucasian, Hispanic and African-American SLE patients [8,9] while GLADEL study group demonstrated the influence of the multi-nationality and ethnicity among SLE patients in Latin America towards the disease outcome [12]. However, the data on morbidity and burden of SLE in Asia is still lacking and majority of the studies which addressed this issue were mainly from Orientals or Chinese ethnicity [13–15].

Malaysia is a multi-racial Southeast Asian country which comprises of three major ethnic groups. The largest ethnic composition is Malay, followed by Chinese and Indian. The catchment area of Universiti Kebangsaan Malaysia Medical Centre (UKMMC) and Putrajaya Hospital comprised of Malays (45.9%), Chinese (43.2%) and Indians (10.3%) [16].

Previous studies which were performed in a tertiary centre in Kuala Lumpur, Malaysia have demonstrated that the prevalence of SLE was seen to be higher in Chinese population, followed by Malays. On the other hand, prevalence of SLE among Malaysian Indians are generally low ranging from 7–11.6% only [17,18]. This was in contrast to Rheumatoid Arthritis in which Indians were predominantly affected as the reported prevalence of RA among them were 54.5% [19].

Although SLE was less common among Indians in Malaysia, they have lower survival rate as compared to other ethnicities [17]. However, this study was done almost two decades ago and the trend may have changed, parallel with the progress in the overall lupus management and care. Herein we described the differences in the disease damage pattern among our multi-ethnic SLE cohort and highlighted the factors contributing to irreversible damage among them.

## Methodology

This was a cross-sectional study involving SLE patients attending a regular follow up and monitoring at the Rheumatology and Nephrology/SLE Clinic in National University of Malaysia Medical Centre (UKMMC) and Putrajaya Hospital, Malaysia. Consecutive patients with minimum disease duration of 6 months were enrolled from August 2013 until January 2015. All patients fulfilled at least 4 criteria from the American College of Rheumatology Classification Criteria for SLE 1997 [20]or renal biopsy consistent with lupus nephritis (LN). The local ethic committee of National University Malaysia Medical Centre and Ministry of Health, Malaysia have approved the study (FF-2013-337 and NMRR-14-386-19203). Informed written consent was taken from the patients and this procedure has been approved by the ethic committee of National University Malaysia Medical Centre and Ministry of Health Malaysia.

### Clinical and laboratory evaluation

Detailed demographic data were collected including age, gender and self-reported ethnicity. Information on the disease characteristics (onset of disease, system involvement in lupus and duration of disease) were obtained from the medical records. The presence of autoantibodies including anti-DsDNA and antiphospholipid antibodies including lupus anticoagulant (LA) and anti cardiolipin (ACL) antibodies were determined from the medical records. The mean cumulative of complement levels (C3 and C4) were determined by calculating the mean of cumulative complement C3 and C4 levels which were done routinely at least 6–12 monthly during their clinical follow-up.

The information on the type of immunosuppressants used at any point of SLE disease course or follow up such as mycophenolic acid (MMF), cyclosporine A (CyA), azathioprine and hydroxychloroquine (HCQ) were determined from the clinical records and electronic pharmacy prescriptions. The history of oral prednisolone ≥1mg/kg daily for over two weeks usage and early HCQ use (less than 3months after SLE diagnosis) was obtained from the medical records. Cumulative exposure to glucocorticoids and cyclophosphamide (CYC) was also calculated. For patients with disease damage (SDI ≥1), the cumulative dose of corticosteroids and CYC were calculated until the date of organ damage development was confirmed. Meanwhile, for patients without disease damage, the cumulative corticosteroids and CYC were calculated until the date of the last visit to the clinic.

### Disease damage assessment

The presence of disease damage was assessed by the rheumatologists and determined from the medical records. Itwas measured using the Systemic Lupus International Collaborating Clinics/American College of Rheumatology damage index (SDI) [2]. This index documents cumulative and irreversible damage, irrespective of its cause, in 12 different organ systems. Apart from damage that is resulted from previous disease activity, the SLICC damage index also measures the irreversible damage due to treatment or medications including corticosteroid induced osteoporosis (CIOP), diabetes mellitus and cataracts. To be scored, each manifestation must be present for at least 6 months. The category of damage and the onset of damage from the date of diagnosis were determined from the medical records. As part of a standard follow up monitoring protocol, a regular bone mineral density scan was performed at least every 2 years in patients who were on long term corticosteroids. Diagnosis of osteoporotic fractures were based clinical history of fracture in the typical sites (neck of femur, spine, wrist) with trivial trauma which was associated with low bone mineral density (T score less than -1). Subsequently, patients were dichotomized into the presence and absence of damage based on a cutoff of the SDI score of 1.

### Statistical analysis

Features from the different parameters were compared between those with disease damage (SDI ≥1) and without disease damage (SDI = 0) using standard statistical tests. All normally distributed numerical data will be expressed as mean ±SD and continuous variables were analyzed with students T test. Non-normally distributed data will be expressed as median ± Inter-quartile range (IQR) and continuous variables were analyzed using Mann-Whitney U test. Chi-square test was used for categorical variables. Significance was taken as p<0.05. Multivariable logistic regression analysis was performed to determine the odds ratio of the each independent predictors of disease damage in LN. All factors which were found to be significantly associated with disease damage (p< 0.05) during the univariate analysis were included in the regression. All statistical analyses were performed using the SPSS program version 22.0.

## Results

A total of 424patients were included into the study. The cohort’s mean age was 38.9 ± 13.5 years with mean disease duration of 9.2 ± 6.8 years. Their mean age at SLEdiagnosis was29.7 ± 13.2 years and majority of them were women (n = 390, 92%). Our cohort consisted of a multi-ethnic populations with predominant Malays (n = 265, 62.5%), followed by Chinese (n = 137, 32.3%), Indians (n = 18, 4.2%), and others (n = 4, 0.9%).

Majority of them had lupus nephritis (n = 272, 64.5%), followed by haematological (n = 264, 62.4%), musculoskeletal (n = 250, 59.4%), mucocutaneous (n = 187, 44.5%), neuropsychiatric lupus (NPSLE) (n = 57 (13.5%) and serositis (n = 45, (10.7%). A total of36 (8.5%) patients had antiphospholipid syndrome (APLS).

### Disease Damage among SLE patients

A total of 182 patients (42.9%) had disease damage (SDI ≥1) with a mean of onset of damage was 5.8 ± 4.8 years from the diagnosis of SLE. The median SLICC/ACR damage index (SDI) was 1 (IQR 1).Table 1 illustrates the system domain of disease damage in the lupus cohort.

**Table 1:** Type and frequency of system damage among the SLE cohort.

Organ/system damage	Frequency (%)
Musculoskeletal	56 (13.2%)
• Avascular Necrosis (AVN)	28
• Osteoporosis	24
• Muscle weakness/atrophy	2
• Osteomyelitis/ septic arthritis	2
Renal	44 (10.4%)
• Chronic Kidney Disease (Glomerular filtration rate < 50%)	28
• End-Stage Renal Disease	15
• Proteinuria	1
Steroid induced Diabetes Mellitus (SDM)	34 (8.0%)
Ocular	26 (6.1%)
• Cataract	19
• Optic atrophy or retinal change	7
Neuropsychiatry	26 (6.1%)
• Cerebrovascular Accident (CVA)	18
• Cognitive Impairement/ Major psychosis	3
• Seizures requiring therapy for 6 months	2
• Transverse myelitis	1
• Peripheral neuropathy	2
Cardiovascular	23 (5.4%)
• Angina/ Myocardial Infarction	16
• Valvular heart disease	6
• Cardiomyopathy	3
Pulmonary	19 (4.5%)
• Pulmonary hypertension	4
• Pulmonary fibrosis	15
Premature gonadal failure	5 (1.2%)
Peripheral Vascular	8 (1.9%)
• Significant tissue loss	4
• Thrombosis	4
Malignancy	7 (1.7%)
Skin	3 (0.7%)
Gastrointestinal resection	2 (0.4%)

Although only 4.2% (n = 18) in our cohort was Indians, however, approximately two-third (n = 12/18, 66.7%) of them had disease damage. This proportion was significantly higher as compared to other ethnicities(42.1%), p = 0.04. Indian patients also have a higher mean SLICC score as compared to other ethnicities. Table 2 illustrates the comparisons of the demographics, disease characteristics, treatment, mean SDI scores and disease damage prevalence between the 3 major ethnics in our cohort.

**Table 2:** Disease characteristics and damage scores by three major ethnic groups.

Variable	Malay (n = 265)	Chinese (n = 137)	Indians (n = 18)	p
Age (years)	37.7 ± 12.8	41.4 ± 15.1	40.1 ± 10.9	0.05
Gender				
Female	91.3 (242)	93.4 (128)	88.9 (16)	0.68
Male	8.7 (23)	6.6 (9)	11.1 (2)	
Disease duration	8.59 ± 6.7	10.0 ± 6.9	10.9 ± 5.7	0.08
SLE system involvement:				
Musculoskeletal	165 (62.7)	71 (51.8)	13 (72.2)	0.06
Mucocutaneous	129 (49)	51 (37.2)	7 (38.9)	0.07
Haematological	166 (62.6)	84 (61.3)	11 (61.1)	0.96
Neuropsychiatric	41 (14.6)	16 (11.7)	0 (0)	0.13
Renal	162 (61.4)	97 (71.3)	10 (55.6)	0.11
Treatment				
Cyclophosphamide, %(n)	43.8 (116)	49.6 (68)	44.4 (8)	0.53
Hydroxycholoroquine, %(n)	74.3 (197)	73.0 (100)	61.1 (11)	0.68
Ciclosporine A, %(n)	28.6 (76)	37.9 (52)	38.8 (7)	0.11
Mycophenolate Mofetil, %(n)	30.2 (80)	35.8 (49)	33.3 (6)	0.49
Azathioprine, % (n)	50.2 (133)	57.7 (79)	50.0 (9)	0.35
Hypertension, % (n)	43.0 (114)	29.2 (40)	33.3 (6)	0.89
Dyslipidaemia,% (n)	28.3 (75)	29.2 (40)	33.3 (6)	0.89
Diabetes Mellitus	8.7 (23)	8.0 (11)	22.2 (4)	0.13
Disease damage	121 (45.7%)	49 (35.8%)	12 (66.7%)	0.02*
SDI scores	0.82 ± 1.0	0.56 ± 0.8	1.3±1.5	0.01*

*p<0.05 = statistically significant

Indian patients predominantly developed steroid-induced Diabetes Mellitus (SDM) as 33.3% of them developed this complication, as compared to only 6.9% from other ethnicities (p = 0.002). Malay ethnicity was associated with corticosteroid induced osteoporosis (CIOP) as 79.2% (n = 19/24) of patient with CIOP were Malays as compared to only 20.8% (n = 5/24) from other ethnicities (p = 0.02). Majority of the osteoporotic fractures occurred at the spinal region (n = 22/24 cases) while two of them had neck of femur fractures. 85.7% (n = 6) of patients who developed malignancy were Chinese while one of them was Malay (14.3%), p = 0.01.

Since our cohort consisted of a minority of Indian ethnicity, comparisons was also made between the two major ethnic groups, ie Malays and Chinese, while excluding the Indian patients. Disease damage prevalence was higher among Malays (n = 121, 45.7%) compared to the Chinese patients (n = 49, 35.8%), p = 0.07. More Malay patients developed CIOP (n = 19,7.2%) compared to the Chinese (n = 4, 2.9%), p = 0.06. Apart from that, pulmonary damage was more also more prevalent among the Malays (n = 16, 6%) as compared to the Chinese patients (n = 3, 2.2%), p = 0.07. Otherwise, there was no significant difference in the prevalence of other disease damage domains between Malays and Chinese patients.

### Factors associated with disease damage in SLE

On univariate analyses, a significantly higher proportion of male patients had disease damage (21/34, 61.8%) as compared to female patients (160/390, 41.0%), p = 0.02. On univariate analyses, presence of disease damage (SDI ≥ 1)was significantly associated with older age, longer disease duration, neuropsychiatry lupus (NPSLE), APLS, positive LA,use oral prednisolone more than 1mg/kg daily over more than 2 weeks, and lower cumulative C3 levels (p<0.05).

Patients who were treated with hydroxychloroquine and received early hydroxychloroquine (HCQ) treatment (started less than 3 months after diagnosis)were less likely to develop disease damage (p<0.01). On the other hand, significantly more patients who had ever received oral prednisolone of ≥1mg/kg daily over 2 weeks had disease damage. Table 3 summarized the demographic and disease characteristics between SLE patients with and without disease damage.

**Table 3:** Demographic, disease and treatment characteristics among SLE patients with and without disease damage.

Parameters	No disease damage, SDI = 0 (n = 242)	Disease damage SDI≥1 (n = 182)	p
Age (mean ± S.D years)	35.8 ± 12.2	43.1±14.2	<0.001
Duration (mean ± S.D years)	7.1± 5.2	11.8 ± 7.7	<0.001
Gender,%			
Female (n = 390)	94.6 (229)	88.5 (161)	0.02
Male (n = 34)	5.4 (13)	11.5 (21)	
Ethnicity,%			
Malay (n = 265)	59.5 (144)	66.5 (121)	0.02
Chinese (n = 137)	36.4 (88)	26.9 (49)	
Indian (n = 18)	2.5 (6)	6.6 (12)	
Others (n = 4)	1.7 (4)	0 (0)	
Age onset (mean ± S.D years)	28.9 ± 12.0	31.1 ± 13.9	0.10
Antiphospholipid syndrome, %	15 (6.2)	21 (11.5)	0.04
Lupus anticoagulant positive, %	13 (5.3)	19 (10.4)	0.05
Anticardiolipin IgG positive, %	57 (23.6)	56 (30.8)	0.10
Anticardiolipin IgM positive,%	40 (16.5)	30 (16.5)	0.90
Anti dsDNA, %	195 (80.5)	133 (73.1)	0.12
Cumulative steroids (mean ± S.D g)	15.2 ± 12.7	12.9 ± 12.3	0.26
Duration steroids (mean ± S.D years)	5.4 ± 4.3	6.1 ± 5.8	0.17
Prednisolon ≥1mg/kg daily, %	45 (18.6)	61 (33.5)	<0.001
Hydroxychloroquine use, %	207 (85.5)	103 (56.6)	<0.001
Azathioprine, %	137 (56.7)	84 (46.2)	0.03
Cyclosporine A, %	77 (31.8)	60 (32.9)	0.43
Mycophenolic acid, %	87 (35.6)	50 (27.5)	0.04
Cyclophosphamide (CYC), %	109 (45.0)	86 (47.3)	0.42
Cumulative CYC (mean ± S.D mg)	2459.9± 6690	1410.5± 2350	0.07
Neuropsychiatric lupus (NPSLE), %	17 (7.0)	40 (21.9)	0.001
Lupus nephritis, %	154 (63.4)	118 (65.2)	0.44
Musculoskeletal, %	145 (63.6)	105 (57.7)	0.72
Mucocutaneous, %	114 (47.1)	73 (40.1)	0.89
Haematology, %	146 (60.3)	118 (64.8)	0.16
Cumulative C3 (mean ± S.D mg/dL)	88.9 ± 24.8	78.6 ± 28.0	0.001
Cumulative C4 (mean ± S.D mg/dL)	18.4±6.9	17.1 ± 7.7	0.10

On multivariable logistic regression analysis, after all significant factors (p<0.05) were included in the analysis, the independent predictors of disease damage among our SLE cohort were age, Indian ethnicity, lower mean cumulative complement C3 level, NPSLE and APLS(all p<0.05). On the other hand, patients who had ever received HCQ and early HCQ treatment were protected against developing disease damage (Table 4).

**Table 4:** Multivariable logistic regression analysis of the independent predictors of disease damage in SLE.

Parameters	B Coefficient	OR (95% C.I)	*P*
Age	0.07	1.07(1.03–1.11)	0.001
Indian ethnicity	3.308	13.541 (1.65–33.9)	0.02
Mean cumulative Complement C3	-.026	0.96 (0.94–0.98)	<0.001
Early Hydroxychloroquine use	-1.29	0.27 (0.14–0.54)	<0.001
Prednisolone ≥1mg/kg daily	1.17	3.22(1.18–8.79)	0.02
Neuropsychiatric lupus (NPSLE)	1.676	5.34 (1.831–15.6	<0.001
Male gender	0.58	1.79 (0.41–7.78)	0.44
Hydroxychloroquine use	-1.22	0.29 (0.10–0.83)	0.02
Mycophenolate Mofetil use	-0.14	0.87 (0.33–2.29)	0.77
Azathioprine use	-0.73	0.48 (0.19–1.18)	0.87
Disease duration	0.05	1.05 (0.96–1.15)	0.24
Antiphospholipid syndrome (APLS)	1.95	6.99 (1.07–45.68)	0.04
Lupus anticoagulant (LA)	0.44	1.56 (0.37–6.46)	0.54

## Discussion

Information on lupus disease progression and damage from South Asian region remains limited and this study is attempted to fill the knowledge gap in this regard. We have presented the prevalence, type and the risk factors of damage in a large multi-ethnic SLE cohort from two Rheumatology centres in Malaysia. Although a direct comparison may not be accurate due to different population selection, disease characteristics (such as disease duration and severity) and study design, our patients demonstrated a comparatively similar prevalence of damage with the two other Asian Chinese cohort [13,14]. In contrast, Pakistani patients were demonstrated to have a markedly high damage rate at 76% but they had longer disease duration of 15 years [21].

The most important finding in our study was that despite Indians were rarely affected by SLE, they were more likely to develop disease damage as compared to other ethnicities. Indians also predominantly develop steroid-induced diabetes mellitus (SDM) complication and this observation can partly be explained by the genetic predisposition to develop insulin resistance among Indian ethnicity [22, 23]. In fact, the prevalence of DM Type 2 among Indians was almost double that of other ethnicities in Malaysia [24]. Our study also demonstrated that Malays tend to develop CIOP with fracture while majority of patients who developed malignancy were Chinese. The predilection of certain ethnicities to develop different types of disease damage need further larger studies as to whether there is a genetic basis to this unique observation remain largely unknown.

The pattern of organ damage in our cohort was similar to those reported for many of other SLE studies, in which musculoskeletal (MSK) was the commonest system affected [10, 13–15,25–32]. Of note, avascular necrosis (AVN) was the most frequent complication that occurred in the MSK domain followed by corticosteroid induced osteoporosis (CIOP) with fractures. This observation was concordant with the Korean and Hong Kong Chinese lupus cohorts [14, 15]. Interestingly, Australian lupus study also found that AVN was more prevalent among their Asian lupus patients [33]. In contrast, many other European [10,32,34] studies reported a more frequent erosive or deforming arthritis. Further investigations were needed to delineate the possible genetic preponderance or other factors associated with the higher risk of AVN among Asians.

Renal involvement and damage were more common in our lupus cohort with no appreciable ethnic differences. The Korean [15]and South Asian Chinese [13, 14]studies reported a lower prevalence of renal damage which was approximately 8%-14.5% despite having a high prevalence of LN. On the other hand, the Pakistani lupus study showed higher renal damage of 37.5% [21]. The discrepancies can be explained by other various factors such as different treatment protocol and a wide spectrum of different histological renal changes which have influence in determining the outcome of LN [35,36].

The striking disparity in the damage pattern in our study was that the prevalence of SDM (18.3%) was higher in our cohort of patients in contrast to other studies which reported the be less than 10%[9–10,13–15,21,27,28]. The pathogenesis of SDM is complex with the interplay of B-cell dysfunction, genetics and lifestyle influences. In our study, it is possible that the steroid use had actually unmasked the underlying DM type 2 as the high prevalence of SDM among our patients was also parallel with the increasing prevalence of DM type 2 among the general population in Malaysia, which is reported to be 22.9% [37].

Various studies have identified factors associated with damage and our results were in agreement with the previous findings that older age [8,15,26,27,30,38], NPSLE [30,31,39] and APLS [40]were associated with damage. Table 5 further illustrates the prevalence of disease damage and the associated risk factors in other SLE cohorts worldwide.

**Table 5:** Prevalence, pattern and factors associated with disease damage among lupus cohorts worldwide.

Study	Country	Patients’ demographics	Damage prevalence	Predictors or Factors of damage
Zonana-Nacach A, 1998 [27]	Mexican	210 patients	117 (55.7%)	Older age Longer disease duration Greater number of ACR criteria at diagnosis Renal involvement Positive anti-dsDNA
Zonana-Nacach A, 2000 [41]	Hopkin’sSLE cohort (USA)	539 patients (Caucasian 52%, African American 46%,Others 2%)	NA	Cumulative prednisone dose was a significant predictor of osteoporotic fractures, coronary artery disease, and cataracts Exposure to high-dose prednisone (≥60 mg/day for ≥2 months) was associated with AVN and stroke
Alarcon GS, 2001 et al [8]	US	258 patients (African Americans 40.3%, Caucasians 31.8%, Hispanic 27.9%)	156 (60.5%)	Age Maximum corticosteroid dose per day Number (ACR) criteria met at baseline Disease activity Abnormal illness-related behaviors.
J Thumboo et al, 2000 [13]	Singapore	69 Chinese	34 (49.3%)	NA
Rivest C., 2000 [25]	USA (5 centers)	200 patients	122 (61%)	African-American race was associated with skin damage Older age at diagnosis correlated with CVS, MSK, GI, ocular and pulmonary damage. Longer disease duration correlated with higher renal and CVS damage Greater disease activity at diagnosis correlated with greater renal, MSK, and pulmonary damage.
Molad et al, 2002 [26]	Israel	151 patients (Jewish 93.4%,Arabs 6.6%)	14 (9.4%) at baseline and 93 (61.6%)at last study visit	Age Prednisone use HCQ protective against damage
Gladman DD, 2003 [28]	Toronto, Canada	73 (87.7% Caucasians)	NA	Corticosteroid therapy
Mok CC, 2003 [14]	Hong Kong Chinese	242 patients	38% at baseline, 55% at year 3	Higher number of major disease flares Use of cyclophosphamide
Toloza SM, 2004 [9]	USA	158 patients (Texan Hispanics 20.3%, Puerto Rico Hispanics 25.3%, African Americans 27.2%, Caucasians 27.2%).	54 (34%)	Hispanic ethnicity from Texas Greater disease activity according to the Systemic Lupus Activity Measure Occurrence of thrombotic events Prednisone at a dosage of < 10 mg/day
Cooper GS et al, 2007 [10]	USA (Carolina Lupus Study)	132 patients (African Americans 53.0%, Whites 47.0%).	80 (61%)	African American ethnicity Lower household income
Sung YK et al, 2007 [15]	South Korea	588 patients	244 (41.5%)	Age Longer disease duration Use of IV cyclophosphamide
Rabanni et al, 2009 [21]	Pakistan	198 patients	150 (75.7%)	NA
Chambers SA, 2009 [34]	UK (1978–2004)	232 patients: Caucasian 168 (72%), Afro-Caribbean 32 (14%), Indo-Asians 23 (10%), Others 9 (4%)	119 (51%)	NA
M. Estévez del Toroet al, 2010 [42]	Cuba	80 patients	39 (48.8%).	Use of higher than 30 mg/day Prednisone doses for more of 4 weeks Leukopaenia Duration of disease
Goncalves et al, 2015 [30]	Portugal	976 patients	363 (37.2%)	Older age Longer disease duration Renal involvement Serositis Neurological involvement Presence of antiphospholipid antibodies Current therapy with steroids
CS Yee et al, 2015 [31]	Birmingham, UK	382 patients (Caucasian 51.6%, South Asian 22%, Afro-Caribbean 20.7%, Chinese 13%).	142 (37.2%)	Higher prior damage Older age at diagnosis Active disease (especially renal and neuropsychiatry) Systemic corticosteroid use Cyclophosphamide use
F Conti et al, 2016 [32]	Sapienza Lupus Cohort, Italy	349 patients (Caucasian 97.1%, Hispanics 2.0%, Asian 0.9%)	125 (35.8%)	Age Disease duration Number of flares Glucocorticoids use

AVN = Avascular necrosis, CVS = cardiovascular, MSK = musculoskeletal.

The use of corticosteroids have been well established to be associated with disease damage [27,29,42,43]. Our study failed to demonstrate any significant association between cumulative and duration of corticosteroid, which were coincide with other studies [32] but the use of high dose oral prednisolone of more than 1mg/kg daily over 2 weeks was a significant predictor of damage. Prednisolone use of more than 30mg daily was also found to be associated with damage in a Cuban lupus cohort [42] while prednisone at the dose of 10-30mg daily was protective against new damage in the LUMINA cohort [9]. However, the use of high dose steroids in our study may also reflect the severity of the disease which also carries higher risk of subsequent damage.

We also demonstrated the early use of HCQ potentially protects against damage and this finding concurred with the Toronto Lupus cohort [44] which showed early protective effect of HCQ among their patients. Antimalarial agents have been used to treat SLE in over 50 years and since 1990s, there is well-established evidence of its various benefits including anti-thrombotic, anti-hyperlipidaemic, prevention of disease flares and damage in lupus [38]. Our previous studies have also found that the use of HCQ was associated with less disease damage in a small lupus nephritis cohort [35] and was associated with less SDM complication [45].

Active disease was an important factor of disease damage in many other previous studies [8, 14, 25]. However, our study did not measure the cumulative disease activity using the standard validated disease activity indices therefore we were unable to demonstrate such association. Nonetheless, low cumulative complement C3 level was demonstrated to be an independent predictor of disease damage and as low C3 is one of the active disease markers, thus this finding further support the notion of high disease activity in predicting damage in SLE. However, since this was a retrospective study and the interval of complement C3 and C4 measurements varied between subjects, therefore, this could lead to bias in our findings.

There were several limitations in our study as some of the other important potential risk factors associated with accrual damage were not evaluated in this study such as cumulative disease activity, flares, and socio-economic status [8, 12]. The health-seeking behaviour and accessibility to the Rheumatology care centres which may also have influence in the outcome of the disease are also not addressed in our study. The retrospective design of our study has a very limited capability to examine the direct causal relationship and the predictors of damage accrual. Notwithstanding these limitations, our study is the first multi-ethnic South Asian lupus cohort which evaluates the association of possible risk factors of damage among patients with SLE. Future prospective studies in large multi-ethnic cohorts should examine this further.

In conclusion, based on this study, the pattern of disease damage in our lupus cohort was slightly unique from other SLE cohorts of other regions with higher percentages of SDM and renal damage observed. The independent factors that were identified to be associated with the disease damage in our SLE patients were age, Indian ethnicity, APLS, NPSLE and mean complement C3 levels. On the other hand, HCQ use and early treatment with HCQ significantly reduces the risk of damage in lupus.

## Supporting Information

S1 Appendix Raw data. (SAV)
